# Do Emotional Faces Affect Inhibition of Return? An ERP Study

**DOI:** 10.3389/fpsyg.2019.00721

**Published:** 2019-04-02

**Authors:** Liping Jia, Jingxin Wang, Kuo Zhang, Hengfen Ma, Hong-Jin Sun

**Affiliations:** ^1^Department of Psychology, Weifang Medical University, Weifang, China; ^2^Academy of Psychology and Behavior, Tianjin Normal University, Tianjin, China; ^3^Department of Social Psychology, Nankai University, Tianjin, China; ^4^College of Foreign Languages, Civil Aviation University of China, Tianjin, China; ^5^Department of Psychology, Neuroscience and Behaviour, McMaster University, Hamilton, ON, Canada

**Keywords:** inhibition of return, emotional faces, attentional bias, event related potentials, lateralised readiness potential

## Abstract

Inhibition of Return (IOR) refers to an individual’s slowed localization or discrimination performance for targets that appear in previously cued versus uncued location after a relatively long delay after cue (∼300–500 ms). The current study adopted a cue-target paradigm and used behavioral and event-related potential (ERP) measures to investigate whether IOR would be modulated by emotional faces during an emotion recognition task. For reaction time measure, we found IOR effect and the magnitude of IOR effect were comparable for fearful face target and neutral face target. For ERP measures, valid cues were associated with smaller P1 and larger N1 waveform than that for invalid cues. Fearful faces were associated with a larger N170 than neutral faces. The onset latency of the stimulus-locked lateralised readiness potential (LRP) in the valid cue condition was longer than that in the invalid cue condition, while there was no significant difference on the onset latency of the response-locked LRP between the valid cue and invalid cue condition. These results support the notion that, regardless the emotion component of the stimulus, the inhibitory bias of attention to previous visited location before response contributes to the IOR.

## Introduction

Considering that you are searching for food, the places you have searched before should be avoided to search again. Such a mechanism that encourages orienting toward only novel location would be useful. Inhibition of return (IOR) is such an adaptive mechanism. IOR describes an attentional cueing effect where participants respond slower to targets in cued locations compared to uncued locations. IOR is typically observed when the interstimulus interval between cue and target are greater than 300 ms – whereas less than 300 ms leads to facilitation effects (Posner and Cohen, 1984). Klein and MacInnes (2010) suggested that IOR improves search processes by inhibiting the search of visited locations. IOR is a “foraging facilitator” that enhances the efficiency during a visual search, and allows people to process targets that appear in novel positions more efficiently. Thus, IOR subserves adaptive behaviors, reflects the flexibility, and adaptive ability of cognitive functioning (Ivanoff and Taylor, 2006). IOR seems to promote visual capture by new locations, increasing the likelihood that the viewer will detect new information.

Classical IOR effect concentrates on the attention modulation for a cued location. Feature of the cue or target seemed to be irrelevant. There are, however, situations in which IOR may conceivably hinder detection of dangerous stimuli. Given that humans show a heightened sensitivity toward emotional information (Everaert et al., 2014; LoBue, 2014; Quiñones-Camacho et al., 2018), these stimuli may escape from the effects of inhibition (Taylor and Therrien, 2005). Therefore, it is reasonable to consider the possibility that stimulus feature might interact with IOR effect. For example, it could be possible that the magnitude of IOR would be affected by the biological significance of the stimuli, such as self-related or emotional stimuli (Stoyanova et al., 2007; Chao, 2010; Gobel et al., 2017). The evidence from different experimental studies on this notion has been mixed.

For the effect of cue feature, Stoyanova et al. (2007) examined whether fearful faces served as the cue would modulate the IOR, they found that IOR disregarded the value of the cue. Regardless the results, some researchers argued that manipulation of emotional valence of the cue might not be the best method to study how the emotional stimuli affected the IOR, as once the salient cue appeared, the attention is inevitably directed toward the exogenous cue, independently of its kind (Baijal and Srinivasan, 2011; Pérez-Dueñas et al., 2014).

Compared to manipulation of cue feature, manipulation of target feature might offer a more meaningful approach as the effect of IOR manifests in the performance for target processing. It has been long established that feature of the search targets themselves affect visual search, for example, a bear can easily be located. Consequently, the searched targets which have an advantage in capturing attention may override the IOR. Therefore it is also important to examine the effect of manipulation of emotional valence of the target in addition to just the location of the cue (Berdica et al., 2017). However, few studies have explored the question of whether IOR can be modulated by biologically important targets.

A recent study examined IOR using twisted self-faces as targets, and suggested that meaningful stimuli on target location could modulate the IOR (Xu and Gao, 2017). Similarly, Taylor and Therrien (2005) used face, scrambled-face and non-face as targets, and asked the participants to detect their location. They found that the magnitude of IOR was unaffected by the target configuration. It was suggested that the mechanism underlying IOR is “blind” to the social significance of visual stimuli, at least when those stimuli carry neutral expressions and are task irrelevant (Taylor and Therrien, 2005). This proposal has received other empirical confirmation (Lange et al., 2008; Wang et al., 2010).

In these studies described above, the task required only localization of target onset, and did not require processing of the target feature. A direct test of feature processing would be through discrimination task (Berdica et al., 2017). Taylor and Therrien (2008) examined the relationship between faces and IOR in a task that required a face/non-face target discrimination. When target configuration was thereby made task relevant, they found that IOR differed for face and non-face targets in terms of magnitude and time course. Studies like Taylor and Therrien (2008) used simplified faces as experimental stimuli (Taylor and Therrien, 2005). While faces in contrast to non-faces might represent biologically significant stimuli, the scrambled faces used as control stimuli might attract even more attention due to their novelty.

In addition to presence of face, emotional signal on the faces could be another important dimension. It has been argued by Mineka and öhman (2002) that mammals possess an evolved fear system, preprogrammed to rapidly detect specific socially threatening facial expressions of others. Fearful faces can be processed with relative automaticity compared to other expressions (Anderson et al., 2003; Stein et al., 2014). Some researchers thought that humans’ brain exerts heightened awareness of fear stimuli compared to other signals (Anderson et al., 2003; Vizueta et al., 2012; Troiani et al., 2014). Very few studies directly test the processing of emotional information in IOR paradigm. A study carried out recently by Silvert and Funes (2016) found that when participants discriminated face from non-face stimuli, their emotional expression had no impact on IOR whatsoever. However, IOR occurred later for fearful versus neutral faces when the participants performed emotion discrimination tasks.

The research on how the biological stimuli affect the IOR can provide important insight into the mechanism of IOR. The original explanation of IOR by Posner et al. (1985) focus on the modulation of attention which is initially involuntarily captured by the cue, then disengaged, and finally inhibited to return to the position previously occupied by the cue (Berlucchi, 2006). This inhibition of attention could be associated with impaired perceptual processes (Prime and Ward, 2006). Recent evidence indicates that IOR may rather result from multiple mechanisms, or from a single mechanism that impacts multiple stages of processing depending on the task parameters (Berlucchi, 2006; Martín-Arévalo et al., 2013).

It is important to point out that the reaction time measure in the studies discussed above might include components from multiple processes, thus might not be able to provide a clear picture of the precise cognition process in the IOR. Event related potentials (ERPs) from the EEG recording, however, could potentially provide supplementary information. Specifically, early components and the motor-related lateralized readiness potential (LRP) recorded in the IOR paradigm could potentially provide a better temporal resolution in separating the contribution of early perceptual processing and later response preparation to overall effect of IOR (Prime and Ward, 2004).

Given the inconsistent results in the literature, it is necessary to explore again the exact influence of biological signal on the targets on IOR in discrimination tasks and provide further evidence on the mechanism of IOR. In the present study, fearful and neural faces selected from Chinese Facial Affective Picture System (CFAPS) (Gong et al., 2011) were served as the targets in the IOR paradigm. The participants were required to discriminate whether the face was a fearful face or a neutral face. In light of the above-mentioned evidences, we hypothesized that fearful faces that appeared in the target place would be more likely to attract attention, which should lead to a smaller IOR compared to neutral faces. In addition to behavioral measure, ERPs were recorded to investigate the mechanism of IOR. For example, early ERP components such as P1 and N1 would reveal the process of attention in the early sensory stage. N170 component has been linked to facial processing, and previous studies have shown changes to the N170 that correspond with different emotional valences (Batty and Taylor, 2003; Pizzagalli et al., 2003). In addition, the LRP component would reveal the cognitive processes involved at different phases of the stimulus-response dynamic. The interval between the stimulus onset and the appearance of the LRP is defined as the stimulus-locked LRP (S-LRP), and the interval between the appearance of the LRP and the response behaviour is defined as the response-locked LRP (R-LRP). The LRP difference between valid and invalid cue conditions would reveal the time course of cognitive process associate with the IOR paradigm. These ERP components could potentially offer insight not only for the effect of target emotion on IOR, but also for the mechanisms of IOR in general.

## Materials and Methods

### Participants and Design

A total of 16 right-handed undergraduates (9 females, mean age 23.7 years, range 21–26 years) participated in this study. All participants had normal or corrected-to-normal vision, and no neurological or psychiatric history. None of them had previously participated in a similar experiment. Ethical approval for this study was obtained from the Research Ethics Committee of Tianjin Normal University, and written informed consents were obtained from all of the participants. All the participants were paid for their participation after the experiment. A 2 (cue type: valid cues vs. invalid cues) × 2 (target expression: fearful vs. neutral) within-participants design were carried out.

### Stimuli and Procedure

Participants performed a modified IOR task in which they had to indicate the emotion of the face picture displaying fearful or neutral expressions. The faces were selected from the CFAPS (Gong et al., 2011), all the hair, ears, neck, external characteristics were removed in the CFAPS, and all the faces were white images with the same brightness, contrast and size. We selected 80 emotional faces (40 fearful faces, 40 neutral faces) as the targets of IOR. Forty-five college students revaluated the valance and arousal of all the selected faces. All the faces were revaluated on a 9 point scale with “1” representing “extremely unpleasant,” “5” representing “neutral,” and “9” representing “extremely pleasant” on the valence, and also a 9 point scale with “1” representing “extremely calm,” “5” representing “neutral,” and “9” representing “extremely excited” was tested to revaluate the arousal. The value of the valence and arousal are shown in Table 1.

**Table 1:** The valence and arousal of the faces.

Face type	Valence	Arousal
fearful	2.82 (0.38)	5.23 (0.23)
neutral	4.15 (0.32)	3.66 (0.21)


Independent sample *t*-test showed that the valence of the two type of faces were different significantly, *t*(78) = -16.95, *p* < 0.001, the valence of the fearful faces were lower than the neutral faces (*M_*negative*_* = 2.82, *SD =* 0.38, *M_*neutral*_* = 4.15, *SD =* 0.32), and the arousal of the two type of faces were different significantly too, *t*(78) = 32.34, *p* < 0.001, the arousal of the fearful faces were higher than the neutral faces (*M_*negative*_* = 5.23, *SD =* 0.23, *M_*neutral*_* = 3.66, *SD =* 0.21). Another four neutral faces (two male, two female) were selected for the practice trials.

**FIGURE 1:**
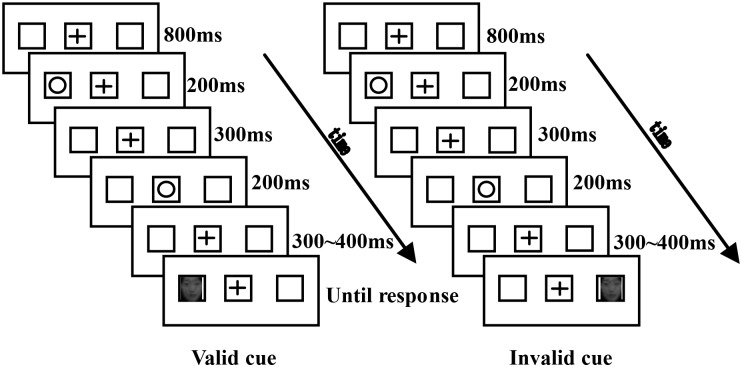
Schematic description of the experimental paradigm. Although the cue is shown inside the left box, it occurred equally often in the left and the right boxes.

A cue-target paradigm was used with the stimulus-onset asynchronies (SOA) of 1000∼1100 msec. The time courses of typical trials in the study are illustrated in Figure 1. Three boxes were presented on the computer screen during the experiment, and a “+” appeared in the middle box for 800 ms as the fixation stimulus. A circle served as the cue would appear equally likely in the left or the right box, lasting for 200 ms. After a 300 ms delay, a circle appeared in the middle box for 200 ms. Then there would be an interval for 300–400 ms with the “+” in the middle box. Lastly, the target (a fearful face or a neutral face) appeared with an equal probability in the left or the right box, and disappeared until the participants made a response. When the face appeared in the cued place, the trial was defined as valid cue. Inversely, the trial was defined as invalid cue. The participants were informed that the cued location would not be predictive of the target location. Participants judged the facial emotion of the target by pressing the “F” key for a fearful face and the “J” key for a neutral face. The response keys were counterbalanced across participants. There was a 1000 ms interval between trials. The size of the picture stimuli were as follows: the fixation point was located at 0.5° horizontal × 0.5° vertical; the facial image in the box was located at 2.34° horizontal × 3.36° vertical; and the perspective angle range of the three boxes was ± 5°.

The experiment was divided into practice and experimental phases. The practice phase contained 24 trials that repeated until participants understood the procedure and reached an accuracy level of 80% or greater. The experimental phase consisted of four blocks with each block including 80 trials. The fearful and neutral faces, as well as the valid and invalid cues were presented in a random order. Participants took a 3 min break between blocks. During the experiment, participants were asked to fix on the “+” sign at the center of the screen and were allowed to blink freely while minimizing actions such as frowning and swallowing.

During the experiment, participants were seated in a dimly lit, soundproofed and electrically isolated room in a comfortable chair, approximately 75 cm from a computer monitor. The E-prime 2.0 were used to record participants’ responses and response times to the IOR task. At the end of the experimental trials, participants were thanked for their efforts, debriefed, and paid by the experimenter.

### EEG Recordings and Signal Processing

This study used E-prime 2.0 to record reaction times and accuracy. Continuous EEG was recorded from 64 cap-mounted Ag/AgCl electrodes arranged according to the 10–20 international placement system (Compumedics Neuroscan, Charlotte, NC, United States), as well as two electrodes placed on the left and right mastoids. Vertical and horizontal eye movements data were recorded with electrodes placed supra- and infra-orbital at the left eye and on the left versus right orbital rim in a bipolar manner. Electrode impedances were maintained below 5 KΩ. Electrical signals were recorded continuously with Neuroscan amplifiers (band pass filter of 0.05–40 Hz) and digitized (sampling rate 500 Hz).

The EEG data was processed and analyzed using Neuroscan 4.3. Off-line, the data was rereferenced to the average activity of the mastoids electrodes and corrected for ocular artifact using the Gratton et al. (1983) method. The EEG data for trials exceeded ± 80 μV (not including eye blink data) were excluded as artifacts. Also the data for trials with erroneous responses were excluded from analysis.

Event-related potentials components were scored by locating the highest peak within predetermined timeframes: P1 (80–110 ms), N1 (110–140 ms), N170 (140–180 ms). Six electrodes (Po5, Po6, O1, O2, Poz, and Oz) were used to detect the P1 and N1 components in the occipital region and two electrodes (P7 and P8) to detect the N170 component in the temporal occipital region.

### Analyses and Results

The data of two participants were excluded from analysis due to excessive EEG artifacts. SPSS 17.0 was used to conduct repeated-measures ANOVAs on the behavioral data and the measurements derived from ERPs waveforms. The Greenhouse-Geisser correction was used to compensate for sphericity violations and *post hoc* tests were Bonferroni corrected. All the dependent variables in different conditions were shown in Table 2.

**Table 2:** All seven dependent variables in different conditions.

	Valid cue		Invalid cue	
Dependent variables	Fearful target	Neutral target	Fearful target	Neutral target
Error rate (ratio)	0.044 (0.022)	0.036 (0.026)	0.039 (0.024)	0.024 (0.016)
Reaction time (ms)	701.54 (24.20)	744.64 (20.09)	667.95 (24.02)	706.89 (17.87)
P1 amplitude (μV)	0.553 (0.427)	0.813 (0.384)	1.099 (0.319)	1.292 (0.290)
N1 amplitude (μV)	-0.872 (0.427)	-1.292 (0.410)	-0.272 (0.318)	-0.227 (0.251)
N170 amplitude (μV)	-2.505 (0.424)	-2.241 (0.378)	-2.159 (0.449)	-1.583 (0.460)
S-LRP latency (ms)	569.43 (3.20)		555.14 (2.11)	
R-LRP latency (ms)	-102.00 (3.98)		-101.07 (3.47)	


*The *SD* were reported in the brackets.*

### Behavioral Results

First we removed outlier data points (less than 100 ms, more than 2000 ms, and more than three standard deviations from each individual’s mean), We then computed the average error rate and the reaction time on the four conditions. A repeated-measures ANOVAs with factors of cue type (valid cues vs. invalid cues) × target type (fearful vs. neutral) were conducted. For the error rate, there was a main effect of cue type, *F*(1,13) = 8.69, *p* < 0.05, *η*^2^ = 0.40, participants gave more error response in the valid trials (*M_error_* = 0.04, *SD =* 0.004) than in the invalid trials (*M_error_* = 0.031, *SD =* 0.003). But the error rate between fearful and neutral targets were not different significantly, *F*(1,13) = 2.15, *p* = 0.17. For the response time, there was a main effect of cue type, *F*(1,13) = 33.84, *p* < 0.01, *η*^2^ = 0.72. As expected, participants responded slower in the valid trials (*M_valid_ =* 723 ms, *SD =* 21) than in the invalid trials (*M_invalid_* *=* 687 ms, *SD =* 20). There was also a main effect of target type, *F*(1,13) = 7.12, *p* < 0.05, *η*^2^ = 0.35, The reaction times for fearful faces (*M_fearful_* *=* 685 ms, *SD =* 24) were significantly shorter than those for neutral faces (*M_neutral_* *=* 726 ms, *SD =* 19). There was no significant interaction between the cue type and target type on error rate and reaction time either, *F_1_*(1,13) = 0.7, *p* = 0.42, *F_2_*(1,13) = 0.21, *p* = 0.65.

### Early ERPs Components

EEGs data were averaged offline for all experiment conditions separately, starting 200 ms before the onset of the target and lasting for 600 ms, generating four ERPs for every subject. The resulting averages were baseline corrected for the 200 ms prior to stimulus onset. The averaged ERPs were low-pass filtered at 30 Hz (24 dB/octave).

**FIGURE 2:**
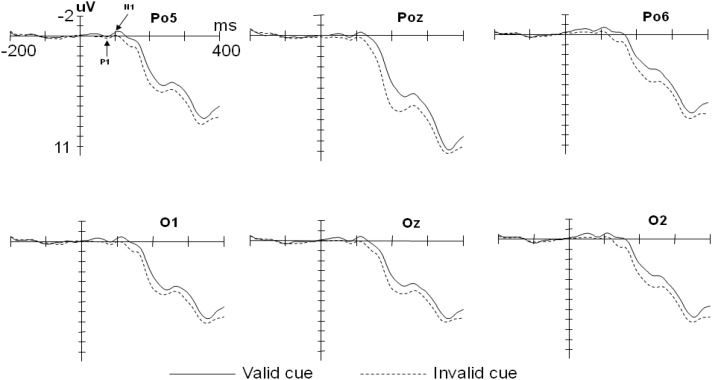
Grand averaged event-related potential (ERPs) illustrating the cue effect.

For the P1 data, A repeated-measures ANOVAs with factors of cue type (valid cues vs. invalid cues) × target type (fearful vs. neutral) × electrode position (Po5, Po6, O1, O2, Poz, and Oz) revealed a significant main effect of cue type on the amplitude, *F*(1,13) = 4.98, *p*<0.05, *η*^2^ = 0.28. The amplitude in the valid cue condition was smaller than that in the invalid cue condition (*M_valid_* *=* 0.68 μV, *SD =* 0.38; *M_invalid_* *=* 1.20 μV, *SD =* 0.24). There was also a significant main effect of electrode position, *F*(5,65) = 5.72, *p* < 0.01, *η*^2^ = 0.31. The amplitudes at Oz and Po5 were larger than those at Po6 and Poz (*M_Oz_* *=* 1.17 μV, *SD =* 0.34; *M_Po5_* *=* 1.01 μV, *SD =* 0.27; *M_Po6_* *=* 0.70 μV, *SD =* 0.25; *M_Poz_* *=* 0.70 μV, *SD =* 0.25) (see Figure 2). The main effect of the target type was not significant, *F*(1,13) = 2.11, *p* = 0.17; The interaction between the cue type and target type was not significant either, *F*(1,13) = 0.012, *p* = 0.913.

For the N1 data, There was a main effect of cue type on the amplitude, *F*(1,13) = 8.76, *p* < 0.05, *η*^2^ = 0.40. The amplitude in the valid cue condition was larger than that in the invalid cue condition (*M_valid_* *=* -1.08 μV, *SD =* 0.40; *M_invalid_* *=* -0.25 μV, *SD =* 0.23). There was also a main effect of electrode position, *F*(5,65) = 3.49, *p* < 0.05, *η*^2^ = 0.21, The amplitudes at O2 and Po6 were larger than those at Poz and O1 (*M_O2_* *=* -0.87 μV, *SD =* 0.28; *M_Po6_* *=* -0.92 μV, *SD =* 0.26; *M_Poz_* *=* -0.45 μV, *SD =* 0.36; *M_O1_* *=* -0.53 μV, *SD =* 0.30) (see Figure 2). The main effect of the target type was not significant, *F*(1,13) = 0.66, *p* = 0.43. No interactions were found for the cue type and the target type, *F*(1,13) = 1.46, *p* = 0.248.

**FIGURE 3:**
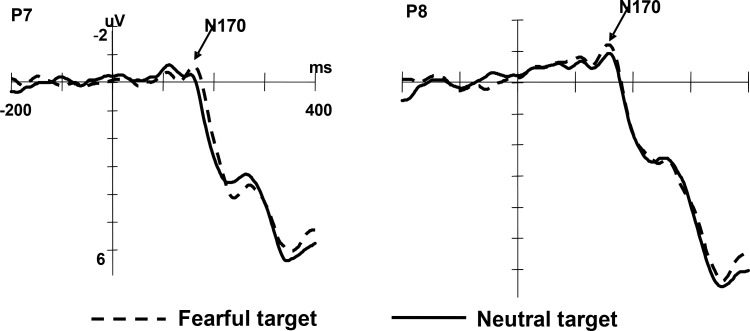
Grand averaged ERPs illustrating emotion effect in two electrodes (P7, left and P8, right).

The N170 elicited by fearful faces was larger than that elicited by neutral faces (*M_fearful_* *=* -2.33 μV, *SD =* 0.40; *M_neutral_* *=* -1.91 μV, *SD =* 0.39), *F*(1,13) = 6.54, *p* < 0.05, *η*^2^ = 0.34. The N170 amplitude under valid and invalid condition was not statistically different, *F*(1,13) = 4.26, *p* = 0.06, *η*^2^ = 0.25. No significant interaction was found, *F*(1,13) = 0.509, *p* = 0.488. These results are shown in Figure 3.

### Lateralised Readiness Potential (LRP)

Lateralised Readiness Potential is a component calculated from C3 and C4 which are located at the scalp near the area of the motor cortex. The LRP provides information concerning covert motor activation that often forms a valuable complement to the information obtained from behavioral measures (Praamstra, 2007). It reveals the cognitive processes involved at different phases of the stimulus-response dynamic. The time course underlying the cognitive processes can be analyzed through measuring the latency of the LRP. In particular, the interval between the stimulus onset and the appearance of the LRP is defined as the stimulus-locked LRP (S-LRP), and the interval between the appearance of the LRP and the response behaviour is defined as the response-locked LRP (R-LRP). In the current study, the onset latency of LRP was set at the point that corresponded to 50% of the area under the LRP waveform (Kisel et al., 2008). The averaged ERPs were low-pass filtered at 30 Hz (48 dB/octave).

**FIGURE 4:**
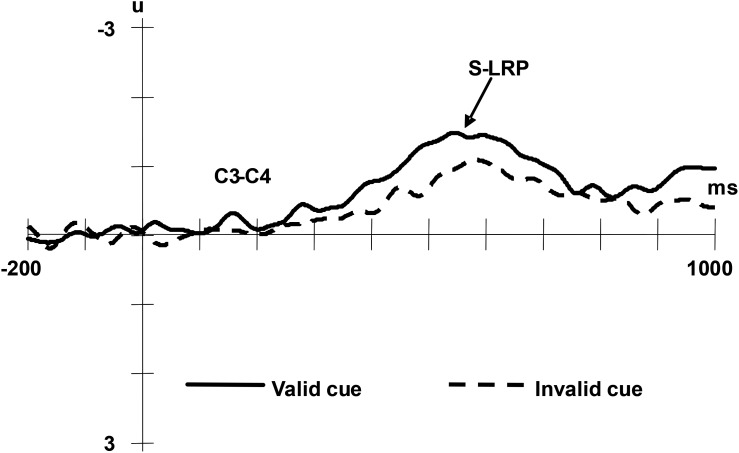
Grand averaged ERPs of the S-LRP under valid cue and invalid cue conditions. The EEG from C3 and C4 were segmented in epochs of 1200 ms beginning 200 ms prior to facial stimulus onset and averaged separately for valid and invalid condition.

The S-LRP was averaged offline separately for valid and invalid condition, starting 200 ms before the onset of the target and continuing for 1200 ms. All the measures were taken relative to the voltage of the 200 ms interval preceding the onset of the target. A paired *t*-test revealed a significant difference between the valid and invalid cue condition with regard to the onset latency of the S-LRP, *t*(13) = 13.67, *p* < 0.01. The onset latency of S-LRP was longer in the valid cue condition compared with the invalid cue condition (*M_valid_* *=* 569 ms, *SD =* 3.2; *M_invalid_* = 555ms, *SD =* 2.1) (see Figure 4).

**FIGURE 5:**
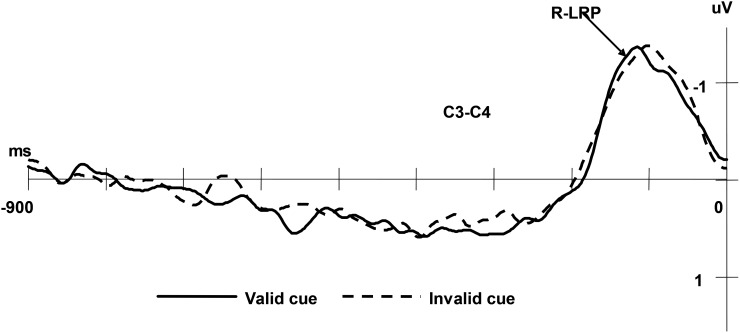
Grand averaged ERPs of the R-LRP under valid cue and invalid cue conditions. The EEG from C3 and C4 were segmented in epochs of 900 ms prior to the response onset and applying –900 ms to –700 ms as the baseline and averaged for valid/invalid condition separately.

The R-LRP was averaged offline separately for valid and invalid condition, starting 900 ms before the response to the targets and continuing for 900 ms. All the measures were taken relative to the voltage of the 200 ms interval after the starting. Paired *t*-tests on the onset latency of R-LRP did not reveal significant differences between the valid and invalid cue conditions, (*M_valid_* *=* 102 ms, *SD =* 4.0; *M_invalid_* = 101 ms, *SD =* 3.5), *t*(13) = -1.96, *p* = 0.07 (see Figure 5).

## Discussion

This study investigated whether IOR would be affected by emotional faces appeared in the target place in a discrimination task. In contrast to our prediction, it was discovered that fearful faces induced attentional bias with the magnitude of IOR comparable to that for neutral faces, demonstrating that emotional targets do not actually influence the IOR effect in a discrimination task. Specifically, behavioral data in this study showed significantly higher error rate and longer reaction time in the valid cue condition compared with the invalid cue condition, which indicated the robust IOR. Although the reaction times for IOR for fearful faces (33.6 ms) were slightly shorter than that for neutral ones (37.8 ms), the difference failed to reach statistical significance, which suggested that the emotional content of the targets did not affect IOR. The present results are consistent with some other studies which previously demonstrated that IOR cannot be alerted by emotion targets (Taylor and Therrien, 2005; Berdica et al., 2017). Previous research supports this stability of IOR even under conditions such as threat or other emotional disorders (Stoyanova et al., 2007; Lange et al., 2008). Wang et al. (2010) also reported no effect of different emotional targets on IOR in discrimination task either, although they did not found main effect of target type (valid vs. invalid conditions) either.

In contrast, Taylor and Therrien (2008) reported larger IOR for faces than that for non-faces. It is possible that the appearance of a face (as oppose to non-face) would create a stronger biological signal compared to the appearance of fearful emotion (as oppose to neutral expression) in our study. But other methodological difference in the two experiments might also contribute to the difference in results. In their study, the participants were asked to discriminate the target as face or non-face. The detection of the presence of the face was relatively easy, which was confirmed by the shorter reaction time in their study. The difference in the magnitude of IOR for face vs. non-face target found in their study may be due to the smaller IOR for the non-face targets because of the novelty of the non-face targets. In our study, instead of detection of the presence of face, our participants were asked to discriminate the emotional categories of the faces which was a more demanding task. We showed that both emotional and neutral faces induced attentional bias but with similar IOR magnitude, indicating that the emotional signal on the targets did not affect the IOR in the difficult discrimination task. Based on recent research (Stoyanova et al., 2007), it is believed that IOR is an adaptive mechanism occurred as a bottom-up processing. In our study, participants were asked to judge the emotional valence of target stimuli, which could be to some extent top-down in nature as the judgment could be influenced by individual experiences. Thus, these processes may occur along different neural circuits independently.

Besides the behavior data, the ERPs results also supported that the IOR was not influenced by the emotional faces appeared in the target position. First of all, the findings showed that N170 amplitude differed between fearful and neutral faces. The N170 component is specific to facial processing, and previous studies have shown changes to the N170 that corresponding with different emotional valences (Batty and Taylor, 2003; Pizzagalli et al., 2003). This research revealed that fearful faces were associated with relatively larger N170 components, which suggested an attentional bias (Luo et al., 2010). The attentional bias to fearful faces is in line with the notion proposed by Silvert and Funes (2016) that IOR should not reduce our chances of noticing event information that could be relevant for our well-being or survival (just as the fearful faces in our study). Furthermore, N170 between valid and invalid conditions were not statistically different, which suggested that the difference for fearful and neutral faces was not different in the IOR paradigm. From these results, it can be concluded that fearful faces can induce attentional bias but the IOR is not influenced by the fearful faces.

Because there was a larger IOR for faces than for non-faces in Taylor and Therrien’s (2008) study, they considered that IOR comes from the response-related processes. However, the reaction time measure could not reveal IOR time course. ERPs used in the present study offer a more sensitive measure of processes involved in IOR paradigm, which may contribute to exploring the mechanism of IOR. Early components P1 and N1 are classic ERPs components identified in visual attention researches (Slagter et al., 2016). Currently, a handful of ERPs studies regarding IOR have revealed differences between P1 and N1 in IOR contexts. Among these studies, the specific findings associated with P1 and N1 differed due to task differences (Luo et al., 2003; Amenedo et al., 2014), and researchers concluded that there was no single neural marker for facilitation and IOR (Martín-Arévalo et al., 2016). In our current study, we found that the valid cue condition yielded smaller P1 amplitudes but larger N1 amplitudes compared with the invalid cue condition. These changes demonstrated that IOR occurs in early sensory stage and that attention plays a role in the IOR process. In addition, in the current study, based on the onset latency for S-LRP and R-LRP, the current study revealed the temporal aspects of cognitive response process, thereby affirming the time when IOR occurs. The longer S-LRP latency for valid cue condition and similar latency of R-LRP for valid vs. invalid cue suggested that IOR affected the process before response but not the motor response. This observation is in line with the notion that the inhibitory biasing of attention contribute to the IOR (Pierce et al., 2017). In short, the current ERPs results illustrated that IOR comes from the process of attention which is a bottom-up process, while distinguishing the target stimuli could be largely a top-down process, therefore in the discrimination task, the emotional stimuli would not influence the IOR.

In conclusion, this study makes a contribution to the debate on whether emotional stimuli influence attentional processes such as IOR. We believe that IOR simply adds an inhibitory tag to the previously attended location to alert individuals that the location has already been searched. In other words, IOR exists as a “foraging facilitator” and is a “blind” attentional mechanism. As a “foraging facilitator,” IOR is extremely robust across different emotional manipulations on the target, which indicates that the purpose of IOR is to enhance the efficiency of visual searches by inhibiting attention toward previously searched locations, and it arises from changes in attention-related processes (Posner and Cohen, 1984).

## Author Contributions

LJ and JW were responsible for the experimental design and drafting the manuscript. KZ was responsible for the data analysis and revising of the manuscript. HM and H-JS were responsible for the interpretation of the data and revising the manuscript.

## Conflict of Interest Statement

The authors declare that the research was conducted in the absence of any commercial or financial relationships that could be construed as a potential conflict of interest.
